# Longitudinal Evolution of the Concentration of Gangliosides GM3 and GD3 in Human Milk

**DOI:** 10.1007/s11745-014-3943-2

**Published:** 2014-09-04

**Authors:** Francesca Giuffrida, Isabelle Masserey Elmelegy, Sagar K. Thakkar, Cynthia Marmet, Frédéric Destaillats

**Affiliations:** Nestlé Research Center, Nestec Ltd, P.O. Box 44, Vers-chez-les-Blanc, 26, 1000 Lausanne, Switzerland

**Keywords:** Ganglioside, Glycosphingolipid, Sialic acid, Human milk

## Abstract

It has been reported that dietary gangliosides may have an important role in preventing infections and in brain development during early infancy. However, data related to the evolution of their concentration over the different stages of lactation are scarce. Liquid chromatography coupled with electrospray ionization high resolution mass spectrometer (LC/ESI-HR-MS) has been optimized to quantify the two major ganglioside classes, i.e., aNeu5Ac(2-8)aNeu5Ac(2-3)bDGalp(1-4)bDGlcp(1-1)Cer (GD3) and aNeu5Ac(2-3)bDGalp(1-4)bDGlcp(1-1)Cer (GM3) in human milk. Gangliosides were extracted using chloroform and methanol, further purified by solid-phase extraction and separated by reversed-phase liquid chromatography. Repeatability, intermediate reproducibility, and recovery values were assessed to validate the method. In human milk, GD3 and GM3 could be quantified at the level of 0.1 and 0.2 μg/mL, respectively, with relative standard deviation of repeatability [CV(r)] and intermediate reproducibility [CV(iR)] values ranging from 1.9 to 15.0 % and 1.9 to 22.5 %, respectively. The described method was used to quantify GD3 and GM3 in human milk samples collected from 450 volunteers between 0 and 11 days and at 30, 60 and 120 days postpartum, providing for the first time the concentration of these minor lipids in a large cohort. The content of total gangliosides ranged from 8.1 and 10.7 μg/mL and the mean intake of gangliosides in infants 30, 60 and 120 days postpartum could be estimated at about 5.5, 7.0 and 8.6 mg of total gangliosides per day, respectively, when infants were exclusively breastfed.

## Introduction

In the first half of the 20th century, newly isolated molecules from ganglion cells of neural tissues were named gangliosides [1]. These molecules are glycosphingolipids formed by a hydrophobic ceramide and a hydrophilic oligosaccharide chain. This chain may contain *N*-acetylneuraminic acid (sialic acid or ‘NANA’ or ‘SA’ or Neu5Ac), or, less commonly, *N*-glycoloylneuraminic acid (Neu5Gc), where a glycol group is bound to the C5 amino group. Structural variations may occur in the lipid moiety of gangliosides; for example, the chain length of the sphingosine base or the fatty acid composition may vary. Gangliosides are widely distributed in almost all human tissues, with the highest amount found in neural tissue and extra-neural organs such the lung, spleen and gut. Variations in ganglioside species are observed based on the type of the tissue; for example, bDGalp(1-3)bDGalNAc[aNeu5Ac(2-3)]bDGalp(1-4)bDGlcp(1-1)Cer (GM1), aNeu5Ac(2-3)bDGalp(1-3)bDGalNAc(1-4)[aNeu5Ac(2-3)]bDGalp(1-4)bDGlcp(1-1)Cer (GD1a), bDGalp(1-3)bDGalNAc(1-4)[aNeu5Ac(2-8)aNeu5Ac(2-3)]bDGalp(1-4)bDGlcp(1-1)Cer (GD1b) and aNeu5Ac(2-3)bDGalp(1-3)bDGalNAc(1-4)[aNeu5Ac(2-8)aNeu5Ac(2-3)]bDGalp(1-4)bDGlcp(1-1)Cer (GT1b) are commonly found in the neural tissue and aNeu5Ac(2-8)aNeu5Ac(2-3)bDGalp(1-4)bDGlcp(1-1)Cer (GD3) and aNeu5Ac(2-3)bDGalp(1-4)bDGlcp(1-1)Cer (GM3) in the extra neural organs [2]. GD3 and GM3 are also the major species of gangliosides in human milk and bovine dairy products. Human colostrum and bovine milk exhibit a unique ganglioside composition, with GD3 as the major constituent [3]. It has been reported [4] that during the first stages of life, dietary gangliosides may have an important role in modifying intestinal microflora and promoting the development of intestinal immunity and oral tolerance in neonates, therefore preventing infections during early infancy. In addition, gangliosides seem to be involved in the processes of tissue and organ development during the neonatal period and in the establishment of neuronal network [5]. During early development and growth of infants, dietary source of gangliosides are human milk, or alternatively, infant nutrition products. Recently, Gurnida et al. [6] concluded that nutritional supplementation with a milk lipid preparation rich in gangliosides appears to have beneficial effects on cognitive development in healthy infants aged 0−6 months.

Several methods were described for the quantitation of gangliosides; however, quantitative data of their concentration in human milk is still scarce [2, 7]. In biological samples, gangliosides are mostly quantified as lipid-bound to sialic acid (LBSA). Sialic acid quantification, with a prior separation of gangliosides and neutral lipids, is usually carried out by using resorcinol-HCl solution [8], which generates a violet-blue color by reacting with sugars. The quantification is performed using a spectrophotometer at 580 nm [8]. The main inconvenience of such colorimetric method is the poor selectivity due to the nonspecific reaction between resorcinol and sialic acid, and also the impossibility of distinguishing between various forms of sialic acid [2]. Sialic acid can also be quantified by high-performance liquid chromatography (HPLC) with or without derivatization. However, these methods supply a gap of sensitivity and they cannot determine individual gangliosides. When the objective is to identify and quantify individual gangliosides, the method includes a Folch-like [9] extraction followed by ganglioside purification using different proportions of chloroform and methanol. Gangliosides are usually separated by high performance thin layer chromatography (HPTLC) and detected by staining with resorcinol. The identification is performed by comparison, running standard mixtures from bovine brain and other species or tissues, and gangliosides are quantified using a densitometer at 580 nm. However, this method is time consuming and it is not convenient for the analysis of large amount of samples. Methods have been developed using specific antibodies against some gangliosides. A variety of monoclonal antibodies recognize specific types of sialic acid and linkage, but only in the presence of specific underlying sugar chains [10]. Recently, quantitative mass spectrometry techniques have been developed [11–15], but none of them, to our knowledge, are aimed at the identification and quantification of GD3 and GM3 molecular species in human milk.

In this study, we describe a validated procedure to quantify gangliosides in human milk using liquid chromatography coupled with electrospray ionization high resolution mass spectrometry (LC/ESI-HR-MS). The method was applied to a large human milk sample set in order to determine the content of gangliosides, providing new insight in infant lipid nutrition.

## Materials and Methods

### Materials

Standard gangliosides GD3 (cat.345752) and GM3 (cat.345733) from bovine milk were purchased from Calbiochem (Merck, Darmstadt, Germany). Methanol (for LC–MS), ammonium acetate (for HPLC) were obtained from Sigma-Aldrich (Steinheim, Germany). Di-sodium-hydrogenophosphate (anhydrous), methanol and water (LiChrosolv) were obtained from Merck (Darmstadt, Germany).

### Methods

#### Human Milk Collection from Mothers

The study took place at Peking University in China. The protocol and collection of human milk was reviewed and approved by the local ethical committee of Beijing. The study was registered in Clinical Trial.gov (NCT01971671). Volunteer mothers of term infants, who were apparently healthy (*n* = 450) provided breast milk samples (~30 mL; between 0 and 11 days and at 30, 60 and 120 days postpartum). Samples were collected after full expression from one breast using a milk pump and while the baby was fed on the other breast to produce a satisfactory let-down in the absence of a suckling response. We made all efforts to collect complete feed that included fore-milk, mid-milk and hind-milk as a representation of one feed, and to avoid within-feed variation of lipid and other nutrient contents. An approximately 30 mL aliquot was separated into two conical 15-mL polypropylene tubes for this study, and the rest was fed to the infant. Samples collected for research were stored at –80 °C and shipped on dry ice to Nestlé Research Center, Lausanne, Switzerland for analysis.

#### Statistical Analysis

A two-sample* t* test assuming unequal variances was applied. Differences are considered significant when the *p* value is below 0.05.

### Gangliosides Identification and Quantification

Ganglioside identification was performed by comparison with the use of both standards, when available, and high scan speed with high resolution (>20,000 FWHM) and high mass accuracy (2 ppm) mass spectrometer (SCIEX 5600 TripleTOF High Resolution Hybrid Mass Spectrometer, Ontario, Canada). Gangliosides were separated by LC using an Aquity BEH C18 column (1.7 µm; 150 × 2.1 mm i.d.; Waters). The chromatography system consisted of Infinity1290 modules (Agilent Technologies, Basel, Switzerland) coupled with high resolution (>20,000 FWHM) and high mass accuracy (2 ppm) mass spectrometer (SCIEX 5600 TripleTOF High Resolution Hybrid Mass Spectrometer, Ontario, Canada). All chromatography was performed at 50 °C. Solvent A composed of water/methanol/ammonium acetate (1 mmol/L) (90/10/0.1 v/v/v) and solvent B of methanol/ammonium acetate (1 mmol/L) (100/0.1 v/v). Gradient conditions were as follows: time = 0 min 10 % solvent A; time = 0.2 min 10 % solvent A; time = 8.2 min 5 % solvent A; time = 12.2 min 5 % solvent A; time = 12.4 min 0 % solvent A; time = 18.4 min 0 % solvent A; time = 18.6 10 % solvent A; time = 21 10 % solvent A. Flow rate was 0.2 mL/min. Injection volume was 0.01 mL for GD3 and 0.005 mL for GM3. The mass spectrometer was equipped with an electrospray ionization (ESI) ion source. The ESI mass spectra were recorded in the negative ion mode under the following conditions: ion spray voltage (IS) –4000 V, temperature of the source 400 °C, declustering potential (DP) –40 V, ion source gases one and two 40 and 35 psi, respectively, curtain gas 15 psi, and collision energy –40 V. GD3 and GM3, were monitored by transitions of the precursor ions to the m/z 290 product ion listed in the Table 1. The ion m/z 290 corresponds to Neu5Ac fragment obtained from B type of cleavage [28]. Data were collected and processed using Multiquant 2.1 and PeakView software (Applied Biosystems, Sciex, Ontario, Canada). Quantification was performed by method of standard addition. Total areas of GD3 and GM3 were calculated as the sums of peak areas of the transitions of the precursor ions to the m/z 290 product ions listed in the Table 1, as previously reported by Sorensen [11]. Only the precursor ions that showed a mass shift lower than 5.0 ppm were quantified. The GM3 saturated species d37:0, d38:0, d40:0, d41:0 and d42:0 were identified with mass shift lower than 5.0 ppm in the standard solution, but not in human milk, probably due to low intensity signal.

**Table 1:** Tentative assignment of human milk gangliosides GM3 and GD3

Type of molecular ion	Predicted (m/z)	Found (m/z)	Mass error (ppm)	Assigned structure
GD3				
[M-2H]^2−^	719.8892	719.8915	3.2	d34:2
[M-2H]^2−^	720.8970	720.8978	1.1	d34:1
[M-2H]^2−^	733.9048	733.9057	1.1	d36:2
[M-2H]^2−^	734.9127	734.9145	2.5	d36:1
[M-2H]^2−^	735.9205	735.9168	5	d36:0
[M-2H]^2−^	741.9205	741.9224	2.5	d37:1
[M-2H]^2−^	743.8892	743.8894	0.3	d38:6
[M-2H]^2−^	745.9048	745.9054	0.8	d38:4
[M-2H]^2−^	746.9127	746.9082	6	d38:3
[M-2H]^2−^	747.9205	747.9222	2.3	d38:2
[M-2H]^2−^	748.9283	748.9303	2.6	d38:1
[M-2H]^2−^	749.9361	749.9331	4.1	d38:0
[M-2H]^2−^	756.9440	756.9434	0.7	d39:0
[M-2H]^2−^	761.9361	761.9373	1.5	d40:2
[M-2H]^2−^	762.9440	762.9466	3.4	d40:1
[M-2H]^2−^	763.9518	763.9487	4	d40:0
[M-2H]^2−^	769.9518	769.9541	3	d41:1
[M-2H]^2−^	770.9596	770.9555	5.4	d41:0
[M-2H]^2−^	775.9518	775.9529	1.5	d42:2
[M-2H]^2−^	776.9596	776.9620	3.1	d42:1
[M-2H]^2−^	777.9674	777.9662	1.6	d42:0
GM3				
[M-H]^−^	1,123.6746	1,123.6770	2.2	d32:1
[M-H]^−^	1,149.6902	1,149.6925	2	d34:2
[M-H]^−^	1,151.7059	1,151.7096	3.2	d34:1
[M-H]^−^	1,173.6902	1,173.6911	0.8	d36:4
[M-H]^−^	1,177.7215	1,177.7227	1	d36:2
[M-H]^−^	1,179.7372	1,179.7418	3.9	d36:1
[M-H]^−^	1,181.7528	1,181.7481	4	d36:0
[M-H]^−^	1,193.7528	1,193.7556	2.3	d37:1
[M-H]^−^	1,195.7684	1,195.7699	1.2	d37:0*
[M-H]^−^	1,199.7059	1,199.7050	0.8	d38:5
[M-H]^−^	1,201.7215	1,201.7212	0.3	d38:4
[M-H]^−^	1,205.7528	1,205.7552	1.9	d38:2
[M-H]^−^	1,207.7685	1,207.7722	3.1	d38:1
[M-H]^−^	1,209.7841	1,209.7858	1.4	d38:0*
[M-H]^−^	1,225.7215	1,225.7219	0.3	d40:6
[M-H]^−^	1,233.7841	1,233.7886	3.7	d40:2
[M-H]^−^	1,235.7998	1,235.8035	3	d40:1
[M-H]^−^	1,237.8154	1,237.8154	0	d40:0*
[M-H]^−^	1,249.8154	1,249.8180	2	d41:1
[M-H]^−^	1,251.8310	1,251.8336	2	d41:0*
[M-H]^−^	1,261.8154	1,261.8177	1.8	d42:2
[M-H]^−^	1,263.8311	1,263.8341	2.4	d42:1
[M-H]^−^	1,265.8467	1,265.8489	1.8	d42:0*

The assignments are based on accurate masses and MS fragmentation. The collision energy (CE) was set at –40 V. Only monoisotopic masses are considered for assignment. Monoisotopic m/z values of ions are given

* Identified with a mass shift lower than 5 ppm in the standard

### Extraction and Purification of Gangliosides

Gangliosides were extracted according to Svennerholm [8], with minor modifications. Briefly, human milk (0.2 mL) was dissolved in water (1 mL) at 40 °C and mixed with 4 mL methanol/chloroform (2/1). The sample solution was shaken and put into an ultrasonic bath. Water (1 mL) was added to the sample solution, which was shaken, put into ultrasonic bath at 25 °C for 10 min. After centrifugation (3,000×*g*, for 10 min), the upper liquid phase was quantitatively transferred into a 15 mL centrifuge tube. The residue was mixed with water (1 mL), 2 mL of methanol/chloroform (2/1), shaken, put into an ultrasonic bath at 25 °C for 10 min, centrifuged (3,000×*g*, for 10 min) and upper liquid phases were pulled together; the volume was adjusted to 12 mL with methanol 60 % and pH to 9.2 by adding Na_2_HPO_4_ 30 mmol/L (0.2 mL). The extract solution was loaded on Oasis HLB VAC RC SPE cartridges (30 mg, 15 mL, Waters) previously conditioned with methanol (2 mL) and methanol 60 % (2 mL). The sample was passed through the cartridge at maximum flow rate of 2−3 mL/min. The sorbent was washed with 2 mL of methanol 60 % and dried by vacuum suction for few seconds; the analyte was eluted with methanol (2 mL). Solvent was evaporated to dryness under a nitrogen flow at 30 °C and the residual lipids were re-dissolved in 0.2 mL of methanol 70 % and stored at –20 °C until analysis.

### Method Validation

In order to account for the matrix effect, a pull of different human milk samples was used to carry out the validation. The method validation is performed to assess the linearity, limit of detection (LoD), limit of quantification (LoQ), trueness and precision. The linearity of the method was assessed by adding to the human milk samples, different concentrations of standard solutions containing from 0.5 to 2.3 and from 1.9 to 9.4 μg/mL of GD3 and GM3, respectively. The calibration curve was plotted as peak areas of GD3 and GM3 vs. concentrations of the standard solutions using a linear regression model. For the quantification of gangliosides, the method of the standard addition was used. The LoD was estimated by signal/noise ratio procedure. Regarding the LoQ, the US Food and Drug Administration (FDA) defines it as the lowest concentration of an analyte in a sample that can be quantitatively determined with suitable precision and accuracy [16]. According to the FDA, the lowest concentration validated should be accepted as the LoQ if the analyte response is at least five times higher than the blank response and if this response is reproducible with a precision of at least 20 %.

Recovery of added standards of GD3 and GM3 was studied at three levels. One milligram of pure standards were accurately weighed and placed in two different 2 mL Eppendorf tubes. After addition of 2 mL of 70 % methanol, the Eppendorf tubes were shaken and standard solutions were added to sample solutions to obtain 0.5, 1.4 and 2.3 μg/mL for GD3 and 1.9, 5.6 and 9.4 μg/mL for GM3. A *t* test was performed to check if recovery of extraction was significantly different from 100 %. The precision of the method was evaluated by calculating the repeatability (r) and the intermediate reproducibility (iR). Repeatability represents the variability of independent results obtained in the same laboratory, with the same analyst, on the same equipment, in a short interval of time. Intermediate reproducibility represents the variability of independent results obtained in the same laboratory, on different days, with the same analyst, different calibrations, and same equipment. Repeatability and intermediate reproducibility were calculated by analyzing spiked samples in duplicate, on seven different days, by the same analyst, with the same equipment and with different solution preparations. Data were evaluated using Q-Stat software (Nestlé, Switzerland).

## Results

### Method Development and Validation

**Fig. 1:**
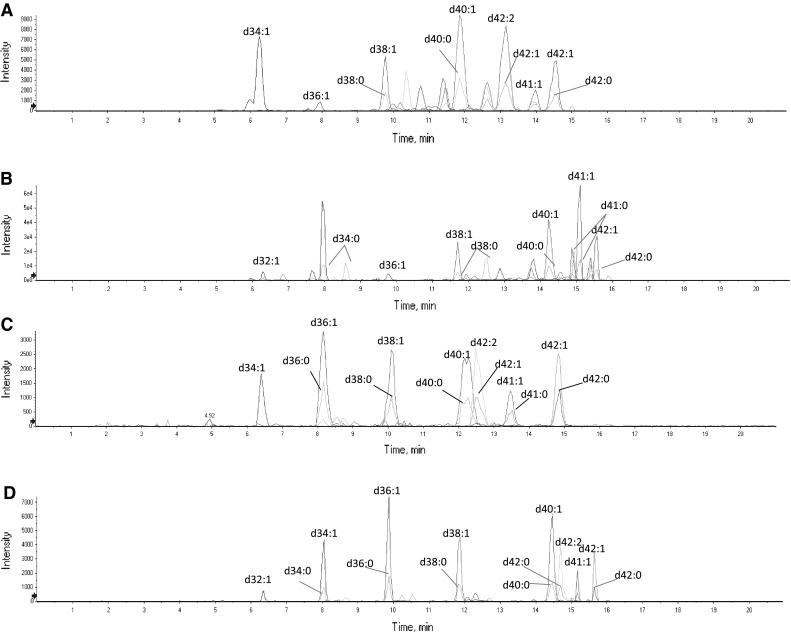
Example of ions chromatogram from MRM scans of main standards GD3 (**a**) and GM3 (**b**) precursor ions and main human milk GD3 (**c**) and GM3 (**d**) precursor ions

Ganglioside molecular species could be separated on reverse phase column according to the oligosaccharide and ceramide portions. Two different columns were tested in this study, including a Luna C5 column (5 μm, 100 × 2.0 mmm I.D.; Phenomenex, Torrance, CA, USA) and an Acquity UPLC BEH C18 column (1.7 µm; 150 × 2.1 mm I.D.; Waters). The Acquity UPLC BEH C18 column had superior resolution compared to the Luna C5 column. MS parameters were optimized by direct infusion of gangliosides standards. Results showed that GD3 and GM3 dissolved in methanol/water (70/30) could generate single charged [M-H]^−^ or double charged [M-2H]^−^ precursor ions with high abundance under negative electrospray ionization. The profile of mass spectra of gangliosides changes according to the chain lengths of fatty acids and sphingosine in the ceramide portion. The molecular species of gangliosides monitored and the mass error are listed in Table 1. Gangliosides were determined by monitoring the product ion at m/z 290 consistent with the elimination of a negatively charged derivate of Neu5Ac [17] and by retention time after having tentatively assigned the molecular species identity by using a high resolution mass spectrometer. Figure 1 shows an example of an ion chromatogram from multiple reaction monitoring (MRM) scans of main ganglioside precursor ions from glanglioside standards and human milk. A similar chromatogram profile was observed between standards and human milk. Among the high intensity ions, GD3 at 734.9127 m/z and GM3 at 1,179.7372 m/z were found to be abundant in human milk and not in the standards. Therefore, it was considered appropriate to relatively quantify gangliosides in human milk using bovine gangliosides as standards. In order to establish the best regression model to quantify gangliosides, the response (in quadruplicate) of four concentration levels covering a range from 0.5 to 10 μg/mL of GD3 and GM3 was measured in human milk. The homogeneity of variance, i.e., dispersion of results at high concentrations, indicated the accuracy of a linear regression model to evaluate results; therefore, a non-weighted linear regression model was used to quantify GD3 and GM3 (results not shown). The LoD was determined by signal/noise ratio procedure and estimated at 0.25 and 0.23 ng on the column for GM3 and GD3, respectively. According to the FDA [16], the lowest validated concentration should be accepted as the LoQ if the analyte response is at least five times higher than the blank response and if this response is reproducible with a precision of at least 20 %. Therefore, LoQ was estimated at 0.47 and 1.88 μg/mL for GD3 and GM3, respectively.

The trueness of the method was evaluated by spiking human milk with gangliosides standards. Recovery was calculated by analyzing spiked samples in duplicate, on seven different days, by the same analyst and with the same equipment. Recovery (Rec) values were compared with reference values. A *t* test was performed to check if recovery was significantly different from 100 %. The recovery values ranged between 97 and 102 % for GD3 and between 100 and 101 % for GM3, and they were not significantly different from 100 %. Finally, the precision of the method was evaluated by calculating the simple repeatability and the intermediate reproducibility. Standard deviation of repeatability [SD(r)] and intermediate reproducibility [SD(iR)], and relative standard deviation of repeatability [CV(r)] and intermediate reproducibility [CV(iR)] are listed in Table 2. CV(r) and CV(iR) values ranged between 1.9 and 15.0 % and between 1.9 and 22.5 %, respectively.

**Table 2:** Median, recovery (Rec), standard deviation of repeatability [SD(r)], relative standard deviation of repeatability [CV(r)], standard deviation of intermediate reproducibility [SD(iR)], and relative standard deviation of intermediate reproducibility [CV(iR)] of GD3 and GM3 in non-spiked and spiked human milk

Analyte	Added amount (μg/mL)	Median	Rec	Rec = 100 %	SD (r)	CV (r) %	SD (iR)	CV (iR) %
GD3	No spiked	0.11	–	–	0.02	15.0	0.02	22.5
	0.47	0.47	102	Y*	0.05	10.1	0.04	8.6
	1.40	1.36	97	Y*	0.18	13.4	0.16	11.4
	2.34	2.34	100	Y*	0.23	9.9	0.16	6.7
GM3	No spiked	0.18	–	–	0.02	8.7	0.03	14.6
	1.88	1.89	101	Y*	0.10	5.4	0.08	4.0
	5.62	5.65	100	Y*	0.11	1.9	0.16	2.8
	9.37	9.37	100	Y*	0.24	2.6	0.18	1.9

Results are expressed in μg/mL of product. Analyses were performed in duplicate by the same operator over 7 days (*n* = 14)

* *Y* = the recovery was 100 %

### Quantitative Analysis of Gangliosides in Full Expressed Human Milk

A total of 450 fully expressed, single breast, human milk samples were analyzed. The samples were collected in a cross-sectional design between 0 and 11 days postpartum and at 30, 60 and 120 days postpartum. Gangliosides differ in the chain length of fatty acids and sphingosine in the ceramide portion. The fatty acid composition of human milk reflects the fatty acid composition of maternal diet [18, 19]; therefore, the profile of gangliosides may change from mother to mother. The gangliosides to be quantified in human milk were selected based on sphingosines d16:0, d18:0, d18:1 and d20:0 with fatty acid side chains of 10−24 carbons. The molecular species of gangliosides monitored and the mass error are listed in Table 1.

**Fig. 2:**
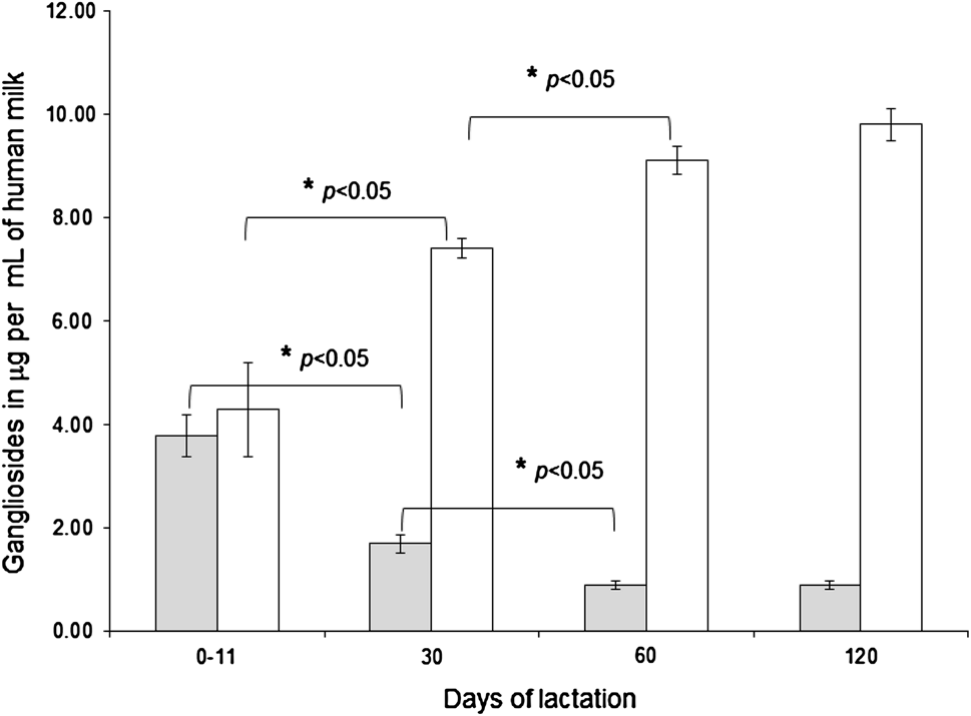
Longitudinal change in the amount of GD3 (in *grey*) and GM3 (in *white*) in human milk. The increase of GM3 concentration and the decrease of GD3 concentration were significantly different (*p* < 0.05) at 30  days postpartum compared with concentrations between 0 and 11 days postpartum, and at 60 days postpartum compared with concentrations at 30 days postpartum. At 120 days postpartum, the GM3 content increased slightly and the GD3 content did not change

The content of GD3 and GM3 in full expressed, single breast, human milk ranged between 0.9 and 3.8 μg/mL and 4.3 and 9.8 μg/mL, respectively (Table 3). In human milk, GD3 is the predominant ganglioside in the early stage of lactation [4]. However, this pattern is quite different in mature milk, in which GM3 represents 50 % of the total gangliosides, while the GD3 content decreases [20–24]. The chronological change of the GM3/GD3 ratio was observed in the analyzed samples, since the GM3/GD3 ratio was one between 0 and 11 days postpartum, four at 30 days postpartum and just over ten at 60 and 120 days postpartum. The amount of GM3 and GD3 was comparable in human milk collected between 0 and 11 days postpartum (4.3 and 3.8 μg/mL, respectively) (Fig. 2). At 30 and 60 days postpartum, GM3 content increased significantly (*p* < 0.05), to 7.4 and 9.1 μg/mL, respectively, and the GD3 content decreased significantly (*p* < 0.05), to 1.7 and 0.9 μg/mL, respectively. At 120 days postpartum, GM3 content increased slightly (9.8 μg/mL) and GD3 content did not change (0.9 μg/mL).

**Table 3:** Ganglioside concentration in human milk samples (*n* = 450) collected between 0 and 11 days, at 30, 60 and 120 days postpartum and expressed in absolute values (μg of gangliosides per mL of human milk)

	0−11 day	30 days	60 days	120 days
GD3 **(**μg/mL**)**	3.8 ± 0.4	1.7 ± 0.2	0.9 ± 0.1	0.9 ± 0.1
GM3**(**μg/mL**)**	4.3 ± 0.9	7.4 ± 0.2	9.1 ± 0.3	9.8 ± 0.3
Total gangliosides **(**μg/mL**)**	8.1	9.1	10.0	10.7
GM3/GD3	1.1	4.3	10.1	10.9

## Discussion

Human milk contains immune factors such as immunoglobulin and lymphocytes [25]. Lower rates of infections-related events, such as necrotizing enterocolitis, meningitis, and urinary tract infection, have been observed in pre-term infants receiving breast milk compared with infant formula [26]. Appropriate amounts of gangliosides may modify the intestinal flora in neonates and have a role in decreasing the infectious capacity of pathogenic bacteria [4, 5]. Breast feeding may be also more important for neurodevelopment compared with infant formula feeding. Wang et al. [27] measured gangliosides in the frontal cortex of infants that had died of sudden infant death syndrome and they report that the infants fed human milk have an average 32 % higher brain ganglioside content (*p* = 0.013) than infants fed infant formula. Recently, Guarnida et al. [6] conducted a study to assess the impact of infant formula supplemented with gangliosides from complex milk lipid, mainly GD3, on cognitive functions of normal healthy infants, showing that supplemented infant formula appears to have beneficial effects on cognitive development functions in healthy infants aged 0−6 months. Therefore, dietary gangliosides may promote the development of intestinal immunity and play a structural and functional role in the brain during early infancy. Despite the fact that several methods were described for the quantitation of gangliosides, quantitative data of their concentration in fully expressed human milk is still scarce [20–24]. The described LC-HR-MS method demonstrated to be sensitive, reproducible and robust for quantifying gangliosides in a large sample set. In addition, the identity of GD3 and GM3 molecular species was tentatively assigned by analyzing the samples with a high scan speed, high resolution (>20,000 FWHM) and high mass accuracy mass spectrometer. To the best of our knowledge, this is the first time that the identification of GD3 and GM3 molecular species has been reported in fully expressed human milk. The lack of pure standards did not allow the absolute quantification of ganglioside molecular species; therefore, further work should be performed to address this issue. Our data confirmed, as previously reported [20–24], that the amount of gangliosides changes during the lactation period, with GD3 decreasing and GM3 increasing over the time. Finally, from this study, it can be estimated that the mean intake of gangliosides in infants 30, 60 and 120 days postpartum was about 5.5, 7.0 and 8.6 mg of total gangliosides per day, if the infant was fed exclusively with human milk. This estimation is based on the assumption that the mean volume of human milk consumed is 600, 700 and 800 mL/day, at 30, 60 and 120 days postpartum, respectively [29].

## Conclusions

In this study, a LC-HR-MS procedure to quantify the most abundant molecular species of GD3 and GM3 in human milk has been optimized and validated. This method has the advantages of selectivity and robustness for the quantification of gangliosides in human milk compared to existing methods. In addition, it can provide details on the molecular species present in ganglioside classes. Finally, the established method was applied to analyze a large number of human milk samples, showing the feasibility of using it for large human clinical trials. The variation in the concentration of gangliosides over the different stages of lactation was observed. However, further investigations are needed in order to determine the biological functions of gangliosides during the early development of infants.

## Abbreviations

CE: Collision energy
CV(iR): Intermediate reproducibility
CV(r): Relative standard deviation of repeatability
FWHM: Full width at half maximum
GD1a: aNeu5Ac(2-3)bDGalp(1-3)bDGalNAc(1-4)[aNeu5Ac(2-3)]bDGalp(1-4)bDGlcp(1-1)Cer
GD1b: bDGalp(1-3)bDGalNAc(1-4)[aNeu5Ac(2-8)aNeu5Ac(2-3)]bDGalp(1-4)bDGlcp(1-1)Cer
GD3: aNeu5Ac(2-8)aNeu5Ac(2-3)bDGalp(1-4)bDGlcp(1-1)Cer
GM1: bDGalp(1-3)bDGalNAc[aNeu5Ac(2-3)]bDGalp(1-4)bDGlcp(1-1)Cer
GM3: aNeu5Ac(2-3)bDGalp(1-4)bDGlcp(1-1)Cer
GT1b: aNeu5Ac(2-3)bDGalp(1-3)bDGalNAc(1-4)[aNeu5Ac(2-8)aNeu5Ac(2-3)]bDGalp(1-4)bDGlcp(1-1)Cer
HPLC: High-performance liquid chromatography
HPTLC: High performance thin layer chromatography
LBSA: Lipid-bound to sialic acid
SA: Sialic acid or Neu5Ac or N-acetylneuraminic acid
LC/ESI-HR-MS: Liquid chromatography electrospray ionization high resolution mass spectrometry
LoQ: Limit of quantification
MRM: Multiple reaction monitoring
Neu5Gc: *N*-Glycoloylneuraminic acid
Rec: Recovery
SD(iR): Standard deviation intermediate reproducibility
SD(r): Standard deviation of repeatability
